# Spontaneous Pneumothoraces and Hemothoraces in Sarcomas

**DOI:** 10.7759/cureus.1905

**Published:** 2017-12-03

**Authors:** Fatima Ezzeddine, Shadia Jalal

**Affiliations:** 1 Internal Medicine, Indiana University School of Medicine; 2 Indiana University Melvin and Bren Simon Cancer Center, Indiana University School of Medicine

**Keywords:** hemothoraces, pazopanib, pneumothoraces, sarcomas

## Abstract

Spontaneous pneumothoraces and hemothoraces are rare manifestations of sarcomas occurring more commonly in specific histologic types, chemotherapy and/or anti-angiogenic therapy. Early identification of spontaneous pneumothoraces and hemothoraces improves the clinical outcomes. In this article, we present a case series of three patients with soft tissue and bone sarcomas who developed spontaneous pneumothoraces and/or hemothoraces and discuss the current literature highlighting the evidence behind these complications.

## Introduction

Primary and metastatic sarcomas have been reported to be associated with spontaneous pneumothoraces and hemothoraces [1-4]. With the emergence and increased use of multitargeted tyrosine kinase inhibitors with anti-angiogenic effects, the risk of developing these complications is even higher [5-7]. We present a case series of the three patients with sarcomas complicated by pneumothoraces and/or hemothoraces followed by a literature review highlighting the evidence behind these complications.

## Case presentation

Case one

**Figure 1:**
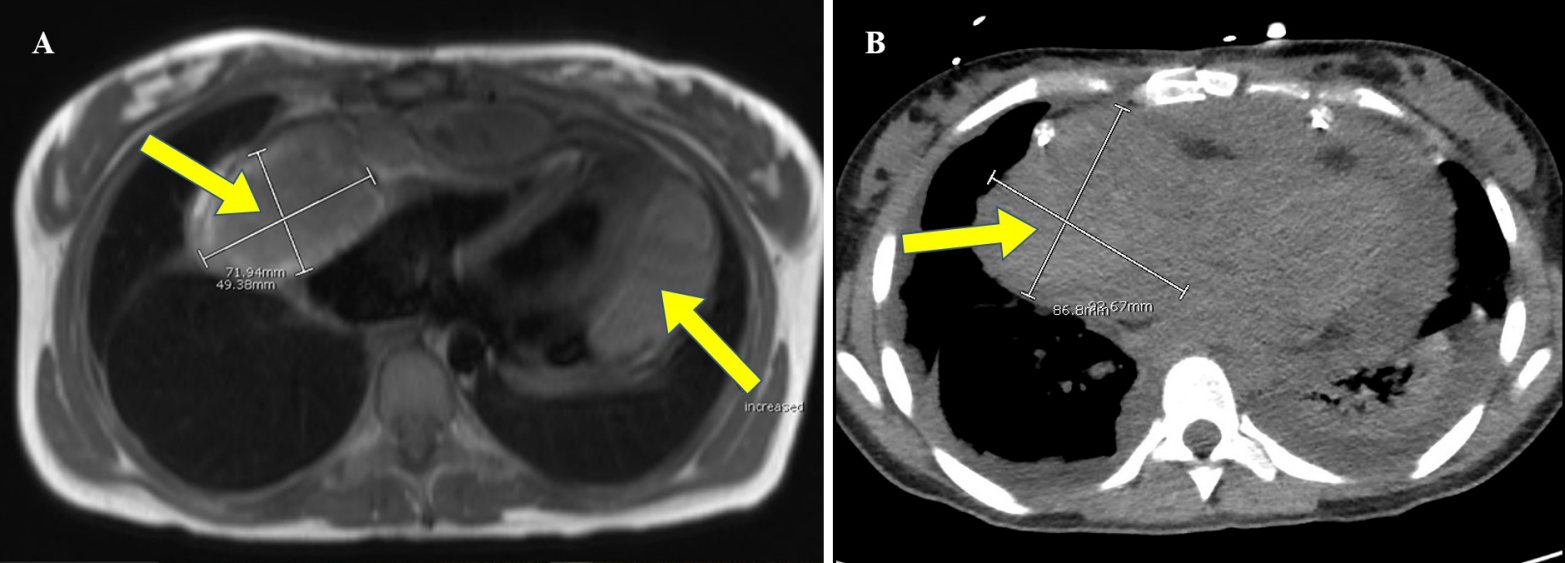
The cardiac magnetic resonance imaging (A) and the computed tomography (B) of the chest illustrating right atrial mass extending into the pericardial sac and a multiloculated fluid collection lateral to the left heart border.

An 18-year-old female presented in December 2015 with acute chest pain. A computed tomography (CT) scan of her chest revealed a pericardial effusion which was negative for malignancy. The presumptive diagnosis was viral pericarditis. In February 2016, she developed recurrent left-sided chest pain and was found to have cardiac tamponade. An enhancing mass was identified in the right atrium on imaging (Figure 1). She underwent a pericardial window procedure which revealed a vascular tumor invading the pericardium. The frozen section of the specimen was consistent with sarcoma. In March 2016, she underwent pericardiectomy with biopsy of the tumor revealing angiosarcoma of the right atrium. After the procedure, she was started on a neoadjuvant cycle of doxorubicin (25 mg/m^2^, one to three days) and ifosfamide (1.8 g/m^2^, one to five days). She was then treated with four other cycles of doxorubicin and ifosfamide followed by four cycles of paclitaxel 80 mg/m^2^ weekly for three of four weeks. Follow up imaging revealed an increase in the tumor size with interval development of small volume metastasis to the right lung. In November 2016, she underwent the surgical resection of the right atrial tumor after she was found to have metastatic lung nodules. The pathology showed angiosarcoma with the treatment effect and evidence of invasion of the cardiac muscle and fibrous connective tissue. New pulmonary nodules were evident on subsequent imaging which led to further chemotherapy treatment. She initially received a cycle of gemcitabine 900 mg/m^2^ followed by four cycles of gemcitabine 1500 mg/m^2^ and dacarbazine 500 mg/m^2^. A week prior to receiving her fifth cycle, she developed severe chest tightness and was found to have left-sided pneumothorax requiring surgical management with left-sided video-assisted thoracoscopic bullectomy. The pathology was negative for metastasis. Currently, she is doing well with plans to resume further chemotherapy.

Case two

**Figure 2:**
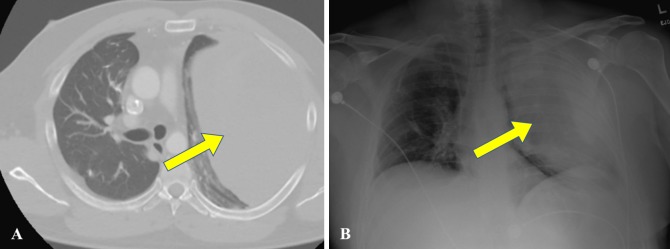
The computed tomography of the chest (A) and x-ray (B) revealing large left-sided hemithorax.

A 39-year-old male reported concerns of progressive right calf swelling for two years. A lower extremity magnetic resonance image (MRI) revealed a mass suspected to be a sarcoma. Fine needle aspirate of the mass was consistent with low-grade monophasic synovial cell sarcoma. A staging CT of his chest showed multiple small bilateral pulmonary nodules. He underwent three cycles of neoadjuvant cyclophosphamide, doxorubicin, and dacarbazine followed by amputation above the right knee. After the surgery, he underwent 10 more cycles of cyclophosphamide, doxorubicin, and dacarbazine with complete resolution of the pulmonary nodules. On follow-up CT of his chest two and a half years later, a left lower lobe metastatic lung nodule was noted. He was re-started with chemotherapy with ifosfamide, mesna, and pegfilgrastim (Neulasta). After three cycles, his chest CT showed disease progression with bilateral pulmonary metastases. The left and right video-assisted thoracoscopy with nodule resections were done, and the surgical specimen pathology was consistent with metastatic synovial sarcoma. At this time, given his progressive disease with chemotherapy, the decision was made to start pazopanib. Two weeks after receiving pazopanib for three months, he was admitted for chest pain and was found to have a pneumothorax which was managed by talc pleurodesis. Pazopanib was resumed, and after receiving it for 29 months, it was temporarily stopped due to uncontrolled diarrhea. Four months later, he developed chest tightness while playing golf and was found to have a recurrent hemothorax six months after the initial one (Figure 2). Both hemothoraces required emergent evacuation and blood transfusions. The examination of the pleural fluid was negative for malignancy. After resolution of his second hemothorax, pazopanib was resumed, and currently, he is doing well.

Case three

**Figure 3:**
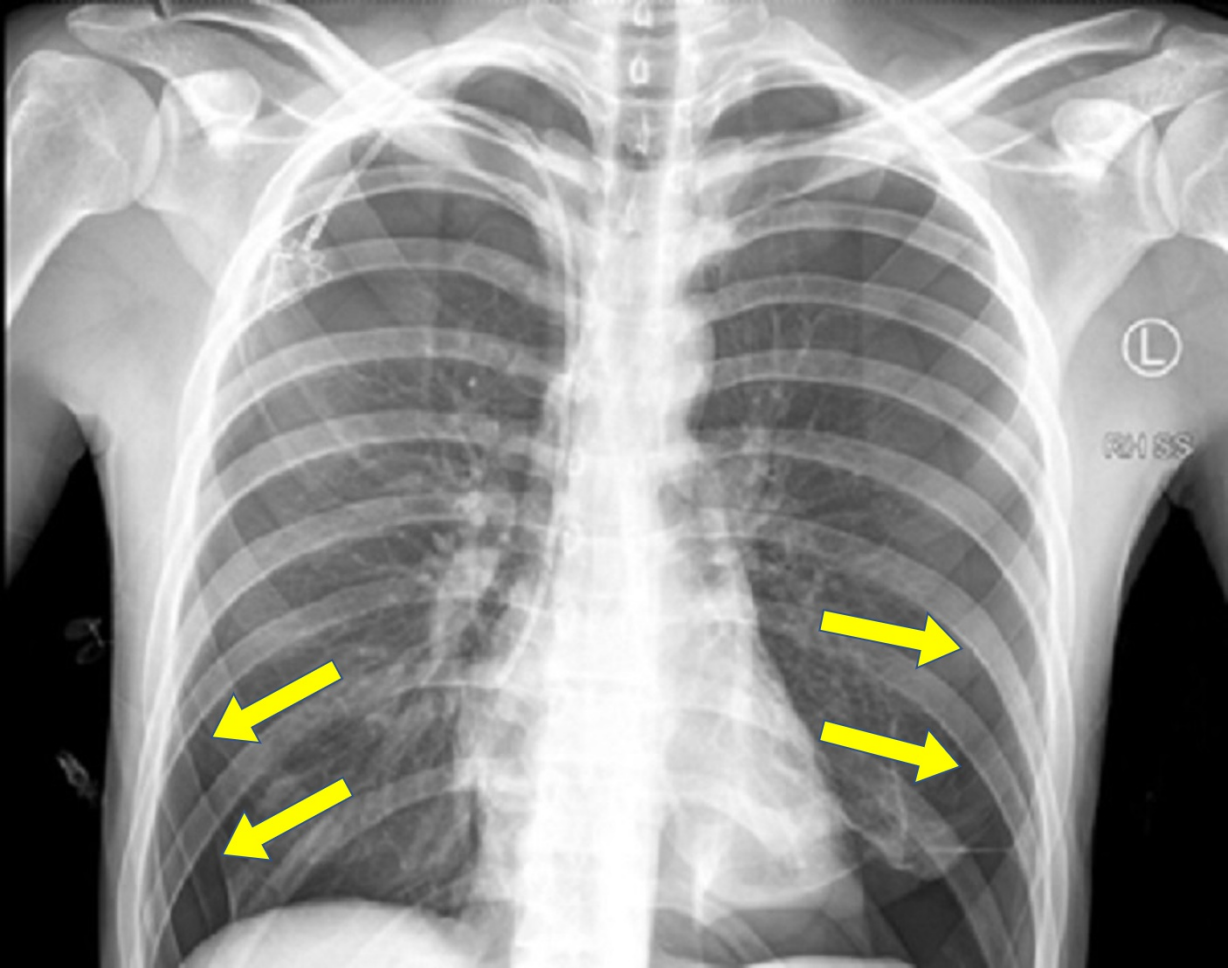
The chest x-ray showing large bilateral pneumothoraces.

A 20-year-old male presented in August 2016 with distal left thigh pain of a few months duration. A left lower extremity MRI revealed distal femoral metadiaphysis mass with surrounding periosteal reaction. The biopsy of the mass revealed osteoblastic osteosarcoma. A staging CT of his chest showed a 3 mm right upper lobe lung nodule. He underwent three cycles of neoadjuvant cisplatin (25 mg/m^2^, one to three days) and doxorubicin (25 mg/m^2^, one to three days). Two weeks after his first cycle, he developed a left pneumothorax, which recurred two weeks later. Given his increased risk for the recurrent pneumothoraces, the pleurodesis was performed. In December 2016, he underwent radical resection of the left distal femur with left total knee arthroplasty and distal femur replacement. The resected tumor showed 50% necrosis. Therefore, the decision was made to pursue three additional cycles of ifosfamide/doxorubicin alternating with ifosfamide/cisplatin. The cumulative doses of ifosfamide, cisplatin and doxorubicin were 27 g/m^2^, 80 mg/m^2^ and 150 mg/m^2^, respectively. Three days prior to receiving his cycle two of ifosfamide/cisplatin, he started to have right-sided chest pain and was found to have bilateral pneumothoraces on imaging (Figure 3). Being hemodynamically stable, he was initially given his planned chemotherapy cycle of ifosfamide/cisplatin followed by chest imaging. A CT of his chest showed three bilateral subpleural cystic lesions which were not demonstrated on prior imaging. He underwent bilateral video-assisted thoracoscopy with lung nodule resection and pleurodesis. The follow-up imaging confirmed the resolution of his pneumothoraces. Currently, he is doing well with plans to perform a chest CT every two months in the first year to detect incidental asymptomatic recurrent pneumothoraces.

## Discussion

Malignancy-related spontaneous pneumothorax is a rare event accounting for 0.05% to 1% of all spontaneous pneumothoraces [1-2]. Its prevalence in soft tissue sarcoma patients is 1.9% [3], while in the patients with primary lung cancers, it is estimated at 0.32% [4]. In 2010, a comprehensive review of spontaneous pneumothoraces complicating sarcoma was published compiling 100 separate articles with a total of 153 patients described [3]. In the Medical Literature Analysis and Retrieval System Online (Medline) and the Excerpta Medica Database (Embase) searches, we have found 21 more articles addressing the same issue with a total of 33 patients which have been published since 2010. We included our three patients (case one, case two, and case three) for a total of 189 patients. The osteogenic sarcoma (31.7%), angiosarcoma (17.5%), and synovial cell sarcoma (11.1%) were the most commonly reported histologies. Furthermore, the chemotherapy and surgery were risk factors for the development of pneumothorax.

Spontaneous hemothorax has also been reported in sarcomas. Our literature review revealed 66 manuscripts reporting hemothorax as a complication of sarcoma with 71 patients described. We included our patient (case 2) for a total of 72 patients. The angiosarcoma cases accounted for around half (51.4%) of the cases. Approximately 50% of the patients had pulmonary involvement, whether primary or metastatic as documented on imaging, surgical biopsy or post-mortem examination. Most cases were initial presentations that led to the diagnosis of cancer. Our patient with metastatic synovial sarcoma developing recurrent hemothoraces following the treatment with pazopanib is the first case noting the occurrence of this complication following pazopanib which might be a cumulative dose effect after receiving it over a prolonged period. In our case, this was preceded by the treatment with pazopanib for 29 months.

With the emergence and increased use of antiangiogenic therapy in the patients with refractory soft tissue sarcomas, the prevalence of pneumothorax complicating soft tissue sarcomas might be higher. Pazopanib is a tyrosine kinase inhibitor that is approved in the United States, European Union, and Japan for the treatment of the patients with refractory or recurrent metastatic soft tissue sarcomas following the chemotherapy. In 2014, the Japanese Musculoskeletal Oncology Group (JMOG) study revealed that the prevalence of pneumothorax in 32 patients treated with pazopanib was 9.4% [5]. The risk factors associated with the development of pneumothorax included: pathological diagnosis of synovial sarcoma, the presence of lung lesions greater than 30 mm in diameter, and a personal history of pneumothorax [6]. Other trials with pazopanib in urothelial cancer, renal cell cancer, pancreatic neuroendocrine tumors and cervical cancer did not report pneumothorax as an adverse event. This might be explained by either underreporting of this adverse effect in other cancer types or the natural course of soft tissue tumors that metastasize predominantly to the lungs with pleural or subpleural involvement.

When examining other anti-angiogenic agents, a phase I trial evaluating the combination of sorafenib, bevacizumab and low-dose cyclophosphamide in children and young adults with refractory solid tumors revealed that 11 of 44 patients (25%) developed pneumothorax potentially related to the therapy [7]. Eight of the 11 patients (72.7%) who developed pneumothorax had pulmonary metastases at the entry of this study. By blocking vascular endothelial growth factor signaling, anti-angiogenic agents disrupt the vasculature of the tumor tissue, and in combination with antiproliferative chemotherapy, they lead to pulmonary nodule necrosis, subsequent rupture of the necrotic nodule, and pneumothorax formation.

## Conclusions

In conclusion, the spontaneous pneumothoraces and hemothoraces are potential complications of soft tissue and bone sarcomas noted at a higher incidence in certain histologic types, following chemotherapy and/or antiangiogenic therapy. Early recognition of primary or metastatic sarcomas as part of their differential diagnosis is likely associated with the improved outcomes. The sarcomas should be in the differential diagnosis of spontaneous hemothorax which can be their initial presentation.
